# From similarity to conceptual—how pictophonetic Chinese characters facilitate inductive reasoning in 5–10-year-old children

**DOI:** 10.3389/fpsyg.2025.1652249

**Published:** 2025-12-02

**Authors:** Xiaoxi Wang, Na Hu, Ruimin Ma, Xiaoying Cai, Xuan Huang

**Affiliations:** School of Preschool Education and Special Education, Kunming University, Kunming, China

**Keywords:** pictophonetic Chinese characters, category, inductive reasoning, semantic radical, phonetic radical

## Abstract

**Introduction:**

Inductive reasoning develops from similarity-based to category-based processes, and linguistic labels are thought to facilitate this shift, though whether they provide similarity or conceptual information remains debated.

**Methods:**

To clarify the role of labels, we examined how Chinese children aged 5–10 interpret pictophonetic characters, focusing on semantic radicals that convey category information. Across three experiments, we manipulated cue strength (no, weak, strong) to test children’s inductive reasoning.

**Results:**

Children relied on phonetic radical similarity in the absence of cues but increasingly favored semantic radicals as cue strength intensified. Younger children tended to reason by perceptual similarity, whereas older children relied more on conceptual information, with evidence suggesting that ages 7–8 mark a developmental turning point.

**Discussion:**

These findings indicate that orthographic awareness and literacy shape the transition from perceptual to conceptual reasoning, and that cue strength significantly influences semantic radical use. The study highlights the educational importance of supporting categorization skills and calls for further research on how character structures contribute to inductive categorization.

## 1 Introduction

Inductive reasoning—the ability to generalize existing knowledge—is the cornerstone of intelligence (Gelman and Markman, 1986; Bhatia, 2024). It is closely related to linguistic information. Research has revealed that linguistic information may affect the processing of hierarchical, category-based induction (Wang et al., 2018; Waxman, 1990) and that linguistic labels may facilitate inductive reasoning (Long et al., 2012).

The Chinese language is built on “characters,” which serve as its most basic functional and grammatical units. A distinctive feature of the writing system is that nearly 80% of commonly used characters have a pictophonetic structure (i.e., they contain both semantic and phonetic radicals). Among these approximately 71% conform to the principle that the semantic radical conveys category information while the phonetic radical indicates pronunciation (Wang et al., 2018). This pictophonetic structure represents a unique orthographic feature of Chinese, distinguishing it from alphabetic systems such as English, where written symbols primarily encode “phonological and semantic information.” In contrast, Chinese characters integrate “graphic, phonological, and semantic information” (Perfetti and Tan, 1998).

A pictophonetic character is composed of two parts: a phonetic radical (sound component) and a semantic radical (meaning component), the latter typically linked to the category attributes of the word. For example, the radical “木” denotes plants. Characters such as 松 (pine), 桃 (peach), and 柏 (cypress) all share this radical and thus belong to the same semantic category. In this way, semantic radicals provide categorical information that supports classification, even when phonetic similarity is absent.

Phonological and semantic processing of Chinese words rely on distinct neural pathways reflecting different cognitive bases for these components (Dong et al., 2005). Building on this, Chen and Zhang (2012) synthesized several studies and proposed a processing-access model for semantic radicals, emphasizing the dynamic interaction between radical processing and whole-character processing. Within lexical and semantic networks, semantic radicals provide specialized channels for accessing word meaning. Supporting this view, Fang and Zhang (2009) found that semantic radicals exert an asymmetric effect: they strongly influence the categorization of Chinese character words, including categorizing speed, but have little effect on naming characters or on naming and categorizing pictures. Notably, characters containing semantic radicals could be categorized as quickly as pictures. Taken together, these findings suggest that semantic radicals may provide an additional “morpho-semantic” pathway from radical form to category membership—one that does not specify exact meaning but does signal relationships among categories.

In evaluating young children’ capacity for inductive reasoning, it is essential to account for the development of language awareness, with particular emphasis on metalinguistic awareness. The development of metalinguistic awareness in children under 12 years can be described in three phases (Gombert, 1992). Pre-emergent (0–3 years): Proto-metalinguistic behaviors appear, such as self-correction of mispronunciations and context-driven lexical simplifications, though their intentionality is debated (Andresen, 1985; Clark, 1978). Acceleration (3–6 years): Rapid, structured growth occurs in phonological (rhyme detection), lexical (polysemy understanding), and syntactic awareness (grammaticality judgments), along with pragmatic adaptations such as the use of politeness markers. This phase is driven by cognitive conflict during rule internalization. Systematization (6–12 years): Literacy enables children to decouple linguistic symbols from referents, supporting orthographic differentiation (e.g., distinguishing homophones) and cross-linguistic analysis in bilinguals. Advanced skills such as logical argumentation (via causal connectors) and metapragmatic humor (e.g., puns) emerge, supported by formal education (Muter et al., 2004; Scarborough and Brady, 2002).

Asian children develop orthographic analysis skills earlier than their English-speaking peers because they learn Chinese characters, while native English speakers tend to focus on phonemic awareness (Pae, 2020). Orthographic awareness emerges during the first years of formal education (Liu et al., 2022). One study found that by age three, children could already distinguish Chinese characters from alphabetic scripts and drawings. Furthermore, by age five, they could differentiate radicals from numbers and judge non-characters with rotated radicals or missing radicals as illegal (Qian et al., 2015). Another study reported that Chinese children demonstrated orthographic awareness by Grade 1, and by Grade 5, their awareness levels were comparable equivalent to those of undergraduates (Li et al., 2000).

The unique structure of Chinese characters is likely to influence Chinese speakers’ category-based inductive reasoning by providing explicit category labels. Long et al. (2012) demonstrated that Chinese linguistic labels can facilitate inductive reasoning. Similarly, Zhang et al. (2014) found that semantic radicals, acting as categorical markers, supply conceptual information through an independent pathway, thereby enabling age-appropriate inductive inferences.

Thus, nouns containing semantic radicals are more conducive to classification. A semantic radical is a natural “label” that marks information about the category of Chinese characters. In the current study, pictophonetic characters were employed to help separate perceptual similarity and conceptual information. Children between the ages of 5 and 10 were selected to study the development of cross-sectional age-related patterns in reasoning abilities. The present study investigates the nature of language labels more precisely by presenting them without corresponding objects, a design that reduces cognitive load for children.

In addition, inductive responses do not depend solely on the availability of category cues. They also require children to inhibit misleading perceptual and phonological matches. Between the ages of 3 and 6 years, inhibitory control shows rapid growth. By the time a child is 7 or 8 years old, most can reliably suppress salient but task-irrelevant information (Davidson et al., 2006; Montgomery and Koeltzow, 2010). This maturational milestone coincides with the emergence of cognitive flexibility and the transition from similarity-bound to rule-based categorization (Zelazo et al., 2003). Therefore, we treat inhibitory control as a latent developmental mechanism: once it reaches sufficient strength (around the time a child is 7–8 years of age), they should be able to override the pull of the phonetic radical and select the semantic one as the basis for induction. The present study examined whether this developmental inflection point is reflected in the shift from phonetic to semantic-radical choices observed in 5–10-year-old Chinese characters.

The age span of 5–10 represent a critical developmental window in which two intersecting trajectories emerge. Cross-cultural research on inductive reasoning shows that the shift from perceptual to category-based generalization consolidates between 6 and 9 years (Gelman and Davidson, 2013; Ralston and Sloutsky, 2023). This transition coincides with rapid growth in inhibitory control and cognitive flexibility, enabling children by ages 7–8 years to reliably override salient but task-irrelevant cues (Davidson et al., 2006; Zelazo et al., 2003).

For children learning Chinese, the same developmental range is marked by qualitative changes in orthographic insights. Large-scale longitudinal studies have shown that explicit awareness of the semantic radical as a morphemic cue emerges around Grade 1 (ages 6–7) and stabilizes by Grade 4 (ages 9–10), at which point radical knowledge predicts independent reading comprehension above and beyond phonological skills (Liu et al., 2022; Qian et al., 2013; Qian et al., 2015). Another study reported that Chinese children demonstrated orthographic awareness by Grade 1, and by Grade 5, their awareness levels were comparable to those of undergraduates (Li et al., 2000). Consequently, the 5–10-year range brackets the transition from “phonetic-dominant” to “semantic-dominant” character processing, providing an ideal test-bed for examining how emerging orthographic knowledge feeds into more general inductive processes. By aligning our tasks with this developmental timetable, we can test whether the 7–8 year inflection point observed in executive control and radical awareness also appears in category-based induction with pictophonetic characters.

**FIGURE 1:**
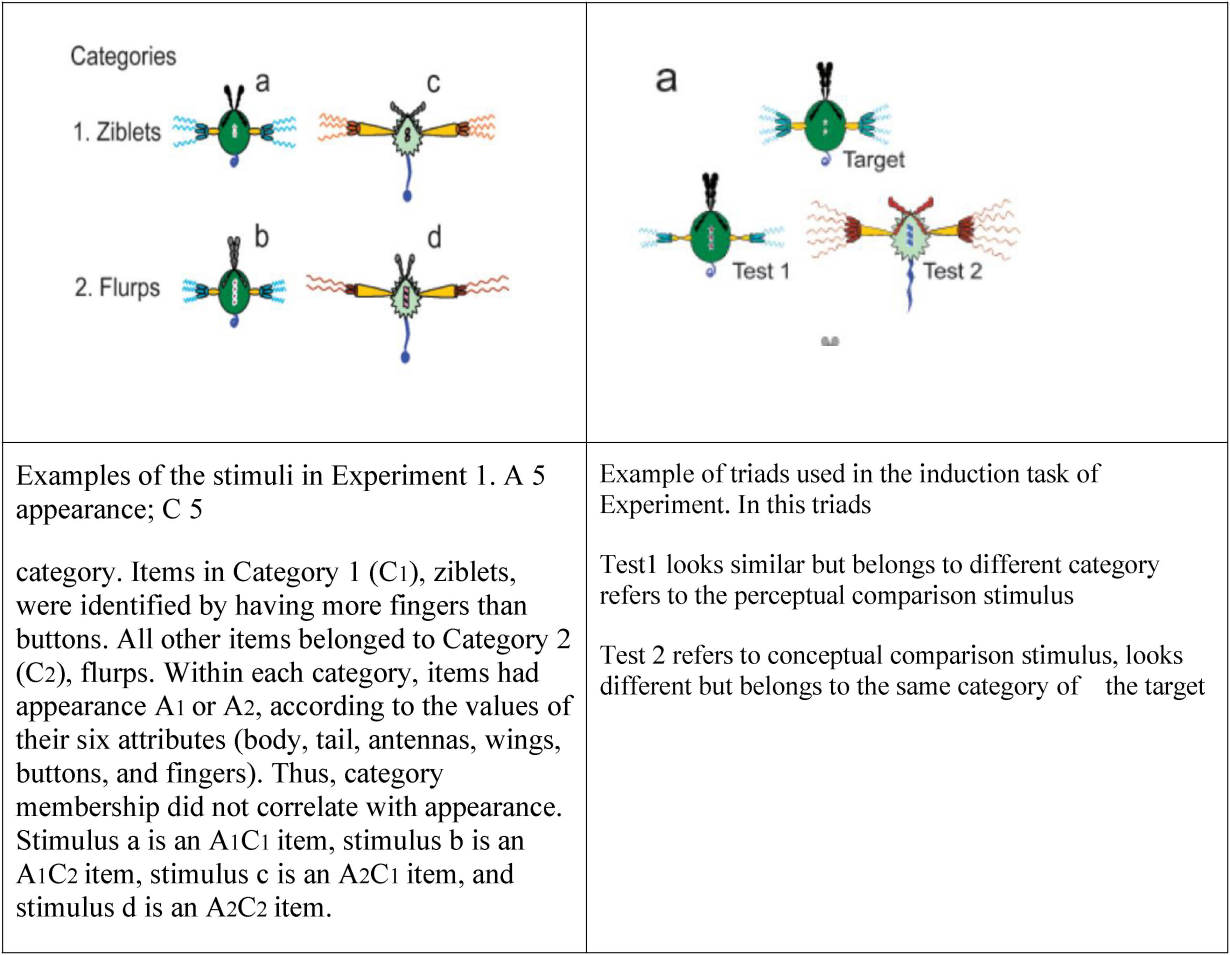
Examples of experimental materials used in triad task (Sloutsky et al., 2007).

This experiment used Chinese characters as the primary material, directly targeting linguistic labels rather than objects such as animals, plants (Long et al., 2012), or man-made artifacts (Sloutsky and Fisher, 2012). In earlier triangulation paradigms (see Figure 1), especially those using artifacts, children first had to match an object to its name before continuing the task. This step was often difficult because artifact recognition does not align well with young children’s cognitive characteristics: artifacts are typically abstract combinations of geometric shapes (triangles, circles, lines, etc.) that are unfamiliar in children’s daily experiences. Thus, their essential features are hard for children to grasp. Consequently, when experiments required children to match pictures to words, the task became unnecessarily complex. By contrast, the present study eliminated this step by focusing directly on words, thereby simplifying the task.

Three levels of cueing intensity were implemented to systematically examine how external prompts influenced children’s use of semantic-radical information in inductive reasoning. First, in the no-cue condition (Experiment 1), target and choice characters were presented without any visual emphasis or explicit explanation of semantic radicals. The children relied solely on spontaneous orthographic knowledge to complete the categorization task. In Experiment 2, we introduced the weak-cue condition, in which the semantic radical of the target character (and the corresponding radical in the SRSC item) was highlighted in red, providing a purely visual attentional prompt. No verbal explanation for the categorical function of the radicals was provided. Finally, in Experiment 3, we introduced the strong-cue condition. In addition to the red highlighting, the experimenter explicitly stated that “the red component indicates the category to which the character belongs,” and this information was accompanied by a brief illustrative example. This constituted a declarative semantic-level prompt. These manipulations allowed us to test whether and how incremental increases in cue salience and semantic explicitness modulated children’s reliance on semantic radicals across age groups.

## 2 Hypotheses and predictions

### 2.1 Experiment 1 (no cue)

In the baseline condition, children were presented with characters without any external cues. This design allowed us to observe their spontaneous reliance on phonetic versus semantic radicals. Based on prior work showing that younger children privilege perceptual similarity while older children increasingly rely on conceptual information, we predicted the following:

#### 2.1.1 H1a Age main effect

Five to six-year-olds, lacking stable semantic-radical awareness, will rely on perceptual similarity, choosing TSPR (same phonetic radical) more often than other options. Seven- to eight-year-olds will show a transitional pattern, with SRSC (same semantic radical and same category) selections increasing but still comparable to TSPR. Nine- to ten-year-olds, with more established form–meaning pathways, and will select SRSC significantly more than TSPR.

- H1b: Relationship-type effects. Because TSPR combines both visual and phonological similarity, it should yield the highest selection rate, whereas SPDC (similar pronunciation, different category) provides only phonological overlap and should yield the lowest.
- H1c Interaction. We expect an Age × Relation Type interaction, such that younger children rely more on TSPR, while older children favor SRSC.

### 2.2 Experiment 2 (weak cue: semantic radical highlighted in red)

Highlighting the semantic radical provides an external attentional cue, which may compensate for immature spontaneous radical awareness in younger children. This manipulation tests whether visual salience alone can shift reasoning toward category-based induction.

- H2a Cue-boost effect. Relative to the no-cue baseline, SRSC selections should increase across all ages, with the largest gain in 5–6-year-olds (external attentional cues compensate for immature.
- H2b The Transition age remains unchanged. Despite the cue, 5–6-year-olds will still perform significantly below the older groups, but the difference between the 7–8- and the 9–10-year-olds will disappear.
- H2c Rank-order shift. The SRSC advantage should eliminate the difference between SRSC and TSPR, while SPDC remains the least selected type.

### 2.3 Experiment 3 (strong cue: red highlighting plus explicit verbal explanation)

By combining visual highlighting with explicit instruction, this condition tests whether direct guidance can accelerate the developmental shift toward semantic-radical use.

- H3a: Instruction-level effect. SRSC will be chosen significantly more often than in the weaker cue conditions, with 5–6-year-olds selecting SRSCs above TSPR for the first time.
- H3b Age-gap reduction. The performance gap between 5- and 6- and 7–8-year-olds will narrow to non-significance, though 9–10-year-olds will still outperform both younger groups (two-tailed).
- H3c Complete reversal. The predicted selection order is SRSC > SRDC > TSPR > SPDC, with SPDC consistently lowest across all three experiments (one-tailed).

## 3 Experiment 1: how the semantic radical facilitates the development of children’s inductive reasoning ability under a no cue condition

### 3.1 Participants

Study participants were recruited from kindergartens and primary schools in Kunming, Yunnan Province, China. A total of 135 participants from three age groups (5–6, 7–8, and 9–10 years) were randomly selected for the study, with 45 participants in each age group. The final valid sample comprised 107 participants, including 34 children aged 5–6 years (mean age: 66.4 months, range: 61–72 months), 33 children aged 7–8 years (mean age: 91.7 months, range: 84–95 months), and 40 children aged 9–10 years (mean age: 115.8 months, range: 109–119 months). This study was conducted in accordance with the principles of the Declaration of Helsinki. The Medical College of Kunming University Medical Ethics Committee approved the human research study. Informed consent was obtained from the parents or guardians of all the study participants. It should be noted that the present work adopted a cross-sectional design: each child was assessed at a single time-point and then assigned to one of three age cohorts (5–6, 7–8, or 9–10 years). Consequently, the data reflect age-related differences across independent groups rather than intraindividual developmental change tracked over time.

### 3.2 Materials

**FIGURE 2:**
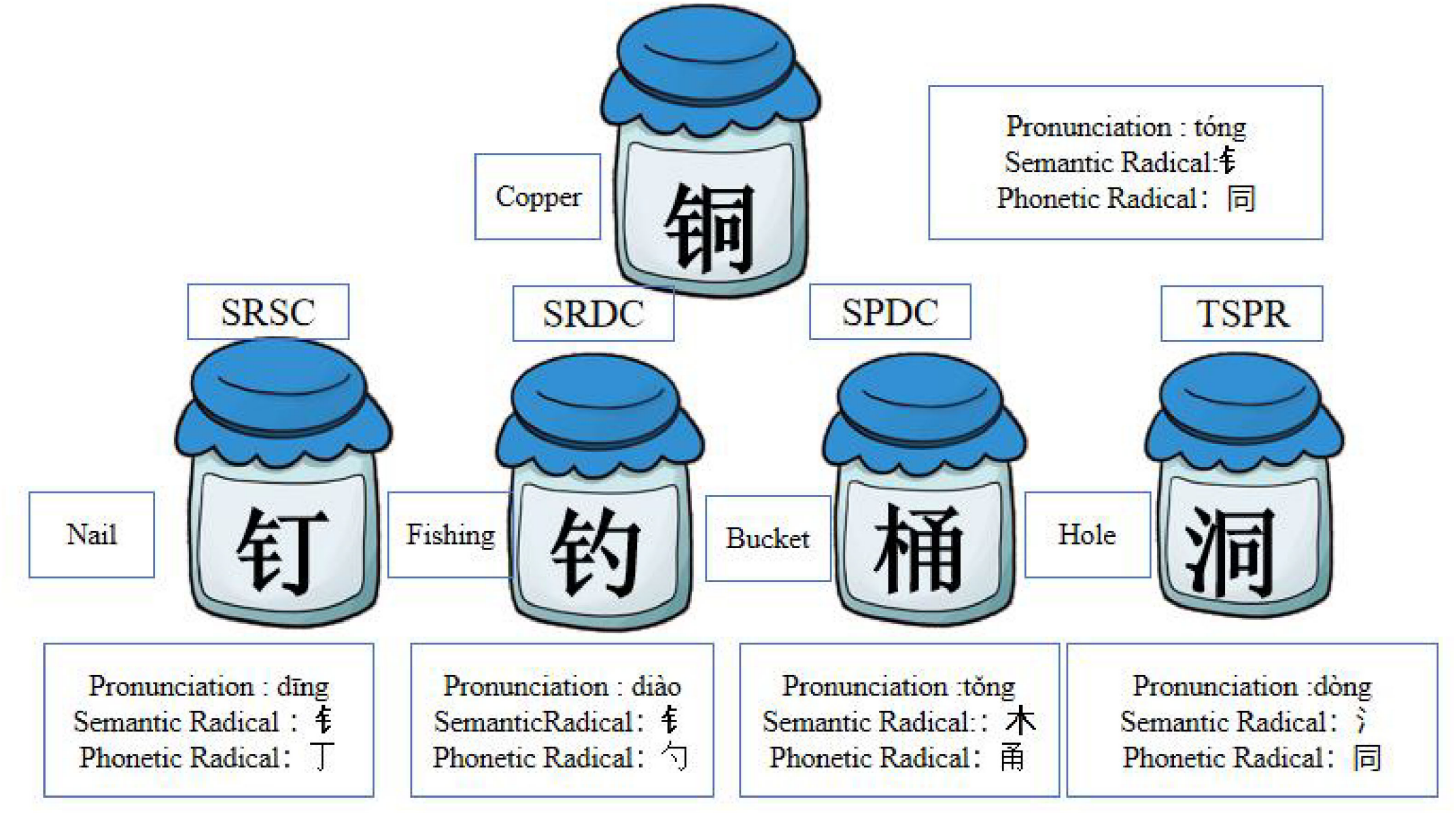
Examples of experimental materials: No cue condition.

Pictophonetic Chinese characters with left-right structures were used as the experimental materials (see Figure 2). Each induction task presented one target character along with four alternative characters, each representing a different categorical relationship to the target.

The fundamental logic of this experimental design is that if children induce according to the consistency of semantic radicals, it indicates their ability to understand categorical information. Conversely, if their selections were based on phonetic similarity, this suggested that their choices were driven by surface similarity. However, because of the inherent characteristics of Chinese characters, many that share phonetic similarity also contain the same phonetic components, that provide visual similarity cues. Therefore, we further differentiated phonetically similar characters into two types: those that had a similar pronunciation but were in a different category (SPDC), and those that had the same phonetic radical that shared both phonetic components and phonetic similarity (TSPR). Regarding semantic radicals, most characters with consistent radicals also share categorical information, although exceptions exist. Therefore, we further refined semantic consistency into two types: (1) semantic radicals that are consistent and share the same categorical information (SRSC), which provide both visual similarity and categorical information; and (2) semantic radicals that are consistent but do not share categorical information (SRDC), which provide only visual similarity. The relationships between the test and target stimuli are summarized in Table 1.

**TABLE 1:** Relationships between test and target stimuli.

Type of test stimuli	Relationship of test and target stimuli			
	Same category	Visual similarity—the same semantic radical	Visual similarity—the same phonetic radical	Auditory similarity
SRSC	√	√		
SRDC		√		
SPDC				√
TSPR			√	√

The semantic radicals of Chinese characters contain categorical information, a unique aspect of orthographic awareness that is gradually acquired during learning. When children are completely unfamiliar with Chinese characters, they treat them as visual images for reasoning. In this case, the consistency of semantic radicals provides only visual similarity, and radicals typically occupy a relatively small portion of the character. If children’s reasoning is based entirely on visual similarity, then the TSPR condition offers the strongest cue (combining both visual and phonological similarity), and its selection rate should be the highest. As children become more familiar with Chinese characters, they begin to recognize that “semantic radicals contain categorical concepts.” Consequently, the selection rates for SRSC and SPDC should increase. With further learning and an expanding repertoire of recognized characters, children should be able to distinguish more precisely between SRSC and SPDC.

### 3.3 Procedures

Prior to the experiment, the multimedia system was connected to the projector, and the test was displayed on the screen. For each trial, five bottles were labeled. The target stimulus was placed centrally in the first row, and the four comparison stimuli were arranged in the second row. The procedure was explained to participants with the following instructions: “Scientists have recently discovered a new thing called “α,” which is so small that only scientists can find it. Your task is to help scientists find this thing.” The researcher then pointed to the target stimulus, read its pronunciation aloud, and told the child it was the name of something that might include “α.” The child was asked to identify which of the comparison stimuli might also contain “α.” The researcher read the alternative words aloud, and the child made a choice. The four relationship types were presented in random positions across trials. Each participant completed 40 trials. The experiment followed a 3 (age group: 5–6, 7–8, and 9–10 years) × 4 (relationship: SRSC, SPDC, SRDC, and TSPR) design.

### 3.4 Results

**FIGURE 3:**
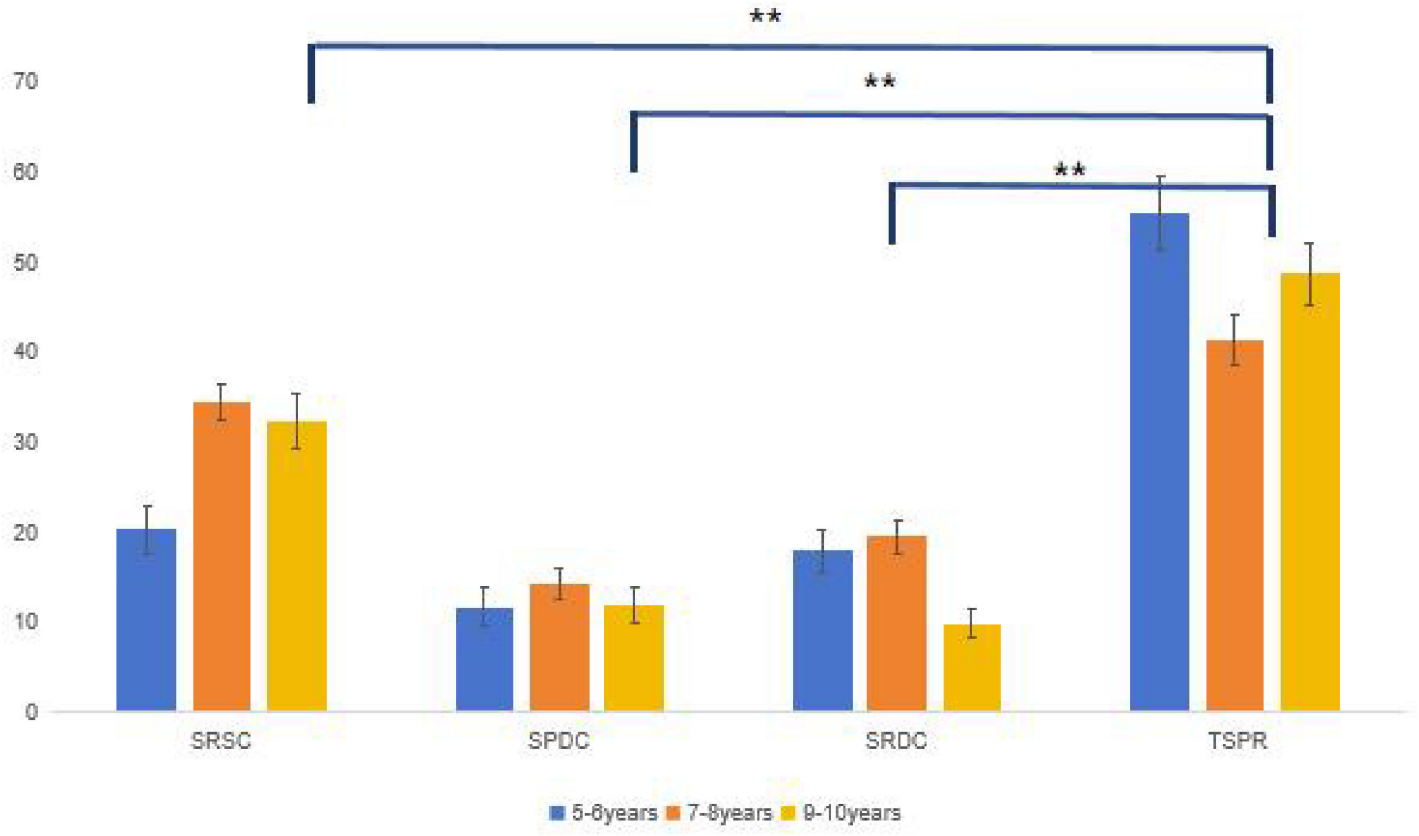
Selection rate in the no cue condition *for *p <* 0.05, **for *p <* 0.01.

We applied an arcsine square root transformation (y $s\,i\,n^{-1}\,\sqrt{p}$) to the percentage data prior to analysis. Accuracy in the test phase was then examined using a 3 (age group: 5–6, 7–8, 9–10 years) × 4 (relationship: SRSC, SPDC, SRDC, TSPR) repeated-measures ANOVA, with relationship as the within-subjects factor. The results are presented in Table 2 and Figure 3.

**TABLE 2:** Results of the no cue condition [M(SE)].

Cue condition	5–6 years	7–8 years	9–10 years
SRSC	20.319(2.63)	34.366(2.015)	32.352(3.073)
SPDC	11.719(2.175)	14.285(1.773)	11.979(1.985)
SRDC	17.896(2.295)	19.562(1.882)	9.904(1.630)
TSPR	55.361(4.079)	41.383(2.712)	48.636(3.497)

SRSC, same radical and same meaning; SPDC, similar pronunciation but no category relationship with target; SRDC, same radical but different category; TSPR, only the same phonetic radical.

There was a significant category relationship main effect [*F*(3, 312) = 89.298, *p <* 0.001, *η_*p*_*^2^ = 0.462]. The Bonferroni-corrected *post-hoc* test revealed that the rate of choosing TSPR was the highest, significantly higher than the choice of SRSC (*p <* 0.001), SPDC (*p <* 0.001), or SRSC (*p <* 0.001). The SRSC rate was the second highest and significantly exceeded those of both SPDC (*p <* 0.001) and SRDC (*p <* 0.001). There was no significant difference between SPDC and SRDC selection rates (*p* = 0.134). There was, however, a significant main effect of age [*F*(3, 104) = 4.311, *p* = 0.016, *η_*p*_*^2^ = 0.077]. The Bonferroni-corrected post-hoc test showed that there was no significant difference between the following age groups: 5–6 and 7–8 years (*p* = 0.225) and 5–6 and 9–10 years (*p* = 0.876). However, there was a significant difference between the 7–8-year and the 9–10-year age groups (*p* = 0.013).

The interaction between relationship and age was significant [*F*(6, 312) = 4.836, *p* = 0.002, *η_*p*_^2^* = 0.085]. The simple effect analysis (for the effect of age under different relationships) showed that the main effect of age was significant in the SRSC [*F*(2, 104) = 7.687, *p* = 0.001], SRDC [*F*(2, 104) = 7.509, *p* = 0.001], and TSPR [*F*(2, 104) = 3.374, *p* = 0.027] categories. However, the difference was not significant under the SPDC category [*F*(2, 104) = 0.477, *p* = 0.622]. SRSC gradually increased with age, with significant differences noted between the 5–6, 7–8-year (*p* = 0.002), and 9–10-year (*p* = 0.005) age groups; however, there was no significant difference between the 7–8 and 9–10-year (*p* = 1.00) age groups. Moreover, there was no age group difference in SPDC (*p =* 1.00). For SRDC, a decreasing trend of use was observed with increasing age, but there were no significant differences between the 5–6 and 7–8-year (*p* = 1.00) age groups. Notably, the ratio of the 9–10-year age group was significantly lower than that of the 5–6-year (*p* = 0.011) and 7–8-year (*p* = 0.002) age groups. The TSPR selection rate in the 7–8-year age group was significantly lower than that in the 5–6-year (*p* = 0.022) group, but not the 9–10-year-old group (*p* = 0.432). There was no significant difference found between the 5–6 and 9–10-year-old children (*p* = 0.513).

A simple effects analysis (examining the effect of relationship type within each age group) revealed significant main effects of relationship for all groups: 5–6 years, *F*(3, 99) = 35.871, *p* < 0.001; 7–8 years, *F*(3, 96) = 26.973, *p* < 0.001; and 9–10 years, *F*(3, 117) = 36.394, *p* < 0.001. For the 5–6-year group, TSPR selections were significantly higher than all other relationships (all *p* < 0.001). SRSC was chosen more often than SPDC (*p* = 0.003), but it did not differ from SRDC (*p* = 1.000). SRDC was selected more often than SPDC (*p* = 0.018). For the 7–8-year group, SPDC was chosen significantly less often than SRSC (*p* < 0.001) and TSPR (*p* < 0.001). No significant difference was found between SRSC and TSPR (*p* = 0.766) or between SPDC and SRSC (*p* = 0.285). For the 9–10-year group, TSPR was chosen significantly more often than SRDC (*p* < 0.001) and SPDC (*p* < 0.001), but not more than SRSC (*p* = 0.090). SRSC was chosen significantly more often than both SPDC and SRDC (both *p* < 0.001). No significant difference was observed between SPDC and SRDC (*p* = 0.090).

In conclusion, all hypotheses were confirmed in Experiment 1. TSPR selection was significantly higher in 5–6-year-olds (55.4%) than in 9–10-year-olds (48.6%), *p* = 0.022. The SRSC increased with age and was significantly higher from 7 to 8 years of age (*p* = 0.002). The interaction was significant, *F*(6, 312) = 4.84, *p* = 0.002, *ηp*^2^ = 0.085.

### 3.5 Discussion

Overall, most children across all age groups chose the same phonetic radical. The rate of choosing the phonetic radical decreased with age, whereas the rate of choosing the same-semantic radical increased with age. It is important to note that the rate of choosing the SPDC was much lower than the rate of choosing the TSPR. Characters with the same phonetic radical usually have similar pronunciations, which provide cues to both visual similarity (same phonetic radical) and phonetic similarity. However, phonetic similarity with different phonetic identities provides cues to phonetic similarity but not to visual similarity; therefore, it does not have the same power inherent in phonetic identity. In addition, the phonetic radical occupies the largest part of a Chinese character, especially for children in the 5–6-year age group. Notably, they commonly selected a phonetic radical when they did not know the character. Further, if the character was identified as a picture, a larger proportion of the parts were consistently grouped together. No significant difference was found between SRSC and SRDC in the 5–6-year age group, which may indicate that 5–6-year-olds regard the character as a picture and use visual similarity in their induction.

During reasoning, a certain amount of inhibitory control is required. As children grow older, their cognitive control gradually develops and matures. They recognize that pronunciation is not directly related to a Chinese character’s meaning and does not capture its core semantic features. Inhibitory control improves rapidly between the ages of 3 and 5 years (Montgomery and Koeltzow, 2010). Cognitive flexibility increases significantly between the ages of 4 and 5 years and is further refined between the ages of 7 and 9 years, reaching relative maturity at the age of 12 years. This study showed that 7–8 years of age marks a turning point in selection patterns, as children shift from relying on surface cues to relying on essential features, consistent with the developmental trajectory of cognitive control outlined in the introduction.

## 4 Experiment 2: how the semantic radical facilitates the development of children’s inductive reasoning ability under the weak cue condition

### 4.1 Participants

Participants were recruited from private kindergartens and elementary schools in Kunming, Yunnan Province. As in Experiment 1, 45 participants from each age group were randomly selected, for a total of 135 participants. To match Experiment 1, 107 valid participants were finally selected, including 34 children aged 5–6 years (mean age: 67.1 months, range: 62–71 months), 33 children aged 7–8 years (mean age: 92.2 months, range: 85–94 months), and 40 children aged 9–10 years [mean age: 116.2 months, range: 109–120 months. The study was approved by the appropriate ethics committee (blinded for review)].

### 4.2 Materials

**FIGURE 4:**
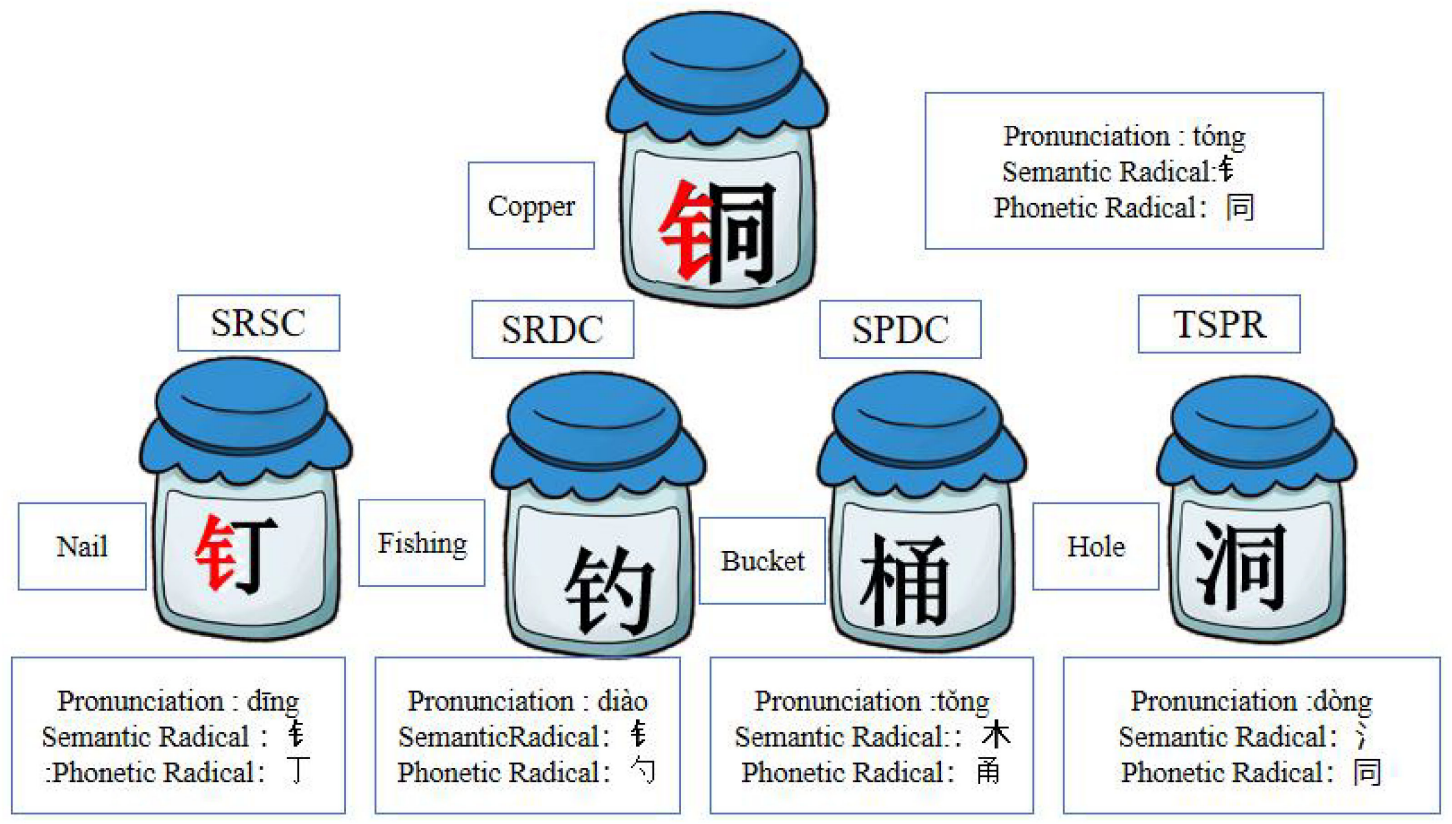
Examples of experimental materials: Weak and strong cue condition.

The experimental materials were the same used in Experiment 1. In this experiment, children were shown a card on which the semantic radical (“钅”) of the target character (铜) and the SPNC (铁) stimulus were marked in red to reinforce visual cues (see Figure 4). After pronouncing the word, the children were asked to select one of four alternatives belonging to the same category as the target character.

### 4.3 Procedures

The instructions were identical to those in Experiment 1. The red highlighting served solely as a visual cue, applied to the target and SRSC characters. No additional prompts were provided.

Experiment 2 used a 3 (age group: 5–6, 7–8, and 9–10 years) × 4 (category relationship: SRSC, SPDC, SRDC, and TSPR) design.

### 4.4 Results

**FIGURE 5:**
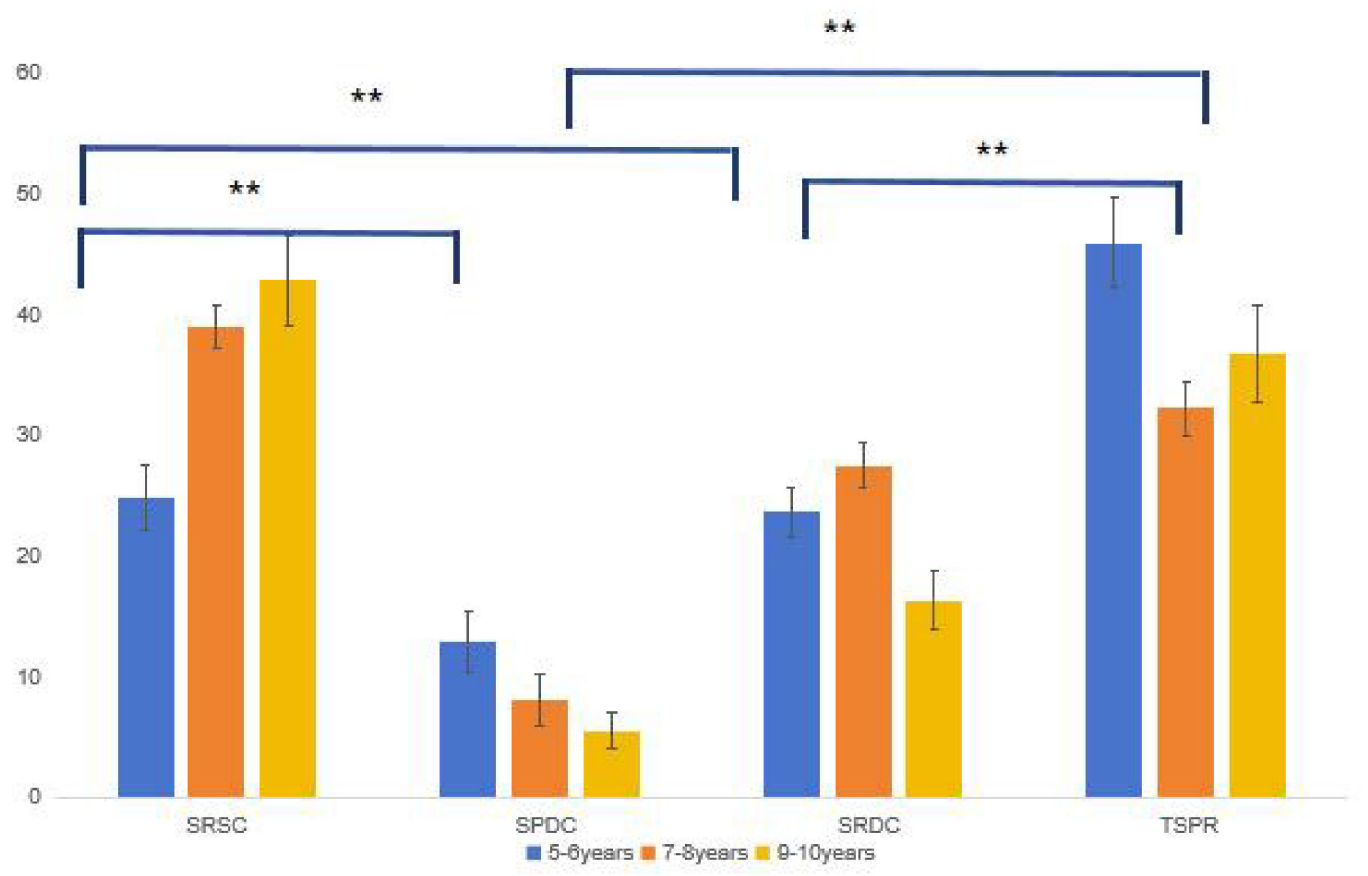
Selection rate in the weak cue condition *for *p <* 0.05, **for *p <* 0.01.

The results of Experiment 2 are presented in Table 3 and Figure 5.

**TABLE 3:** Results of the weak cue condition [M(SE)].

Cue condition	5–6 years	7–8 years	9–10 years
SRSC	24.726(2.73)	38.887(1.814)	42.736(3.805)
SPDC	12.892(2.500)	8.016(2.183)	5.431(1.512)
SRDC	23.575(2.099)	27.398(1.870)	16.252(2.425)
TSPR	45.866(3.702)	32.144(2.280)	36.641(4.018)

SRSC, same radical and meaning; SPDC, similar pronunciation but no category relationship with the target; SRDC, same radical but different category; TSPR, the same phonetic radical.

The relationship main effect was significant [*F*(3, 312) = 54.355, *p* = 0.001, *ηp*^2^ = 0.343]. The *post-hoc* test (Bonferroni correction) showed that TSPR and SRSC had the highest selection rates, with no difference between them (*p* = 1.000). TSPR was chosen significantly more often than SPDC (*p* < 0.01) and SRDC (*p* < 0.01). SRSC was also chosen significantly more often than SPDC (*p* < 0.01) and SRDC (*p* < 0.01). Finally, SPDC was selected significantly less often than SRDC (*p* < 0.01). The age main effect was significant [*F*(3, 104) = 4.176, *p* = 0.018, *ηp*^2^ = 0.074], and the *post-hoc* tests (Bonferroni correction) showed that the difference between the 5–6 and 7–8-year age groups was not significant (*p* = 1.000), nor was the difference between the 7–8 and 9–10-year age groups (*p* = 0.068). However, the difference between the 5–6 and 9–10-year age groups was significant (*p* = 0.032).

The interaction of relationship and age was significant [*F*(3, 312) = 6.029, *p <* 0.001, *ηp*^2^ = 0.104]. A simple effects test (for the effect of age on the different relationships) revealed that the main effect of age was significant for all four relationships: SRSD [*F*(2, 104) = 9.665, *p <* 0.001]; SPDC [*F*(2, 104) = 3.463, *p* = 0.035]; SRDC *F*[2,104) = 6.914, *p* = 0.002]; and TSPR [*F*(2, 104) = 3.732, *p* = 0.027]. Children aged 9—-10 years had the highest preference for SRSC. This was significantly higher than that of children aged 5—-6 years (*p <* 0.001), but not different from that of children aged 7—-8 years (*p* = 1.000). Children aged 7—-8 years also had a higher preference for SRSC than those aged 5—-6 years (*p* = 0.006). The 5–6-year age group chose SPDC significantly more often than the 9–10-year age group (*p* = 0.031), but not more often than the 7–8-year age group (*p* = 0.319). The SPDC selection rate among the 7–8-year age group did not differ from that of the 9–10-year age group (*p* = 1.00). Regarding the SRDC relationship, the highest selection rate was found in children aged 7—-8 years old. This rate was significantly higher than that for children aged 9—-10 years (*p* = 0.001), but not 5–6-year-old children (*p* = 0.704). For the SRDC condition, there was no significant difference found between the 5–6-year-olds and the 9–10-year-olds (*p* = 0.055). In terms of the TSPR relationship, the highest selection rate was among children aged 5–6 years, and this rate was significantly higher than that among children aged 7—-8 years (*p* = 0.027). However, the difference with respect to children aged 9—-10 years was not significant (*p* = 0.189), nor were the differences between children aged 7—-8 years or children aged 9—-10 years (*p* = 1.000).

A simple effects test (examining relationship type within each age group) showed significant main effects in all groups: 5–6 years, *F*(3, 99) = 18.466, *p* < 0.001; 7–8 years, *F*(3, 96) = 32.466, *p* < 0.001; and 9–10 years, *F*(3, 117) = 23.840, *p* < 0.001. For the 5–6-year age group, TSPR was chosen significantly more often than SRSC (*p* = 0.010), SPDC (*p* < 0.001), and SRDC (*p* = 0.001). SRSC was chosen significantly more often than SPDC (*p* = 0.004), but it did not differ from SRDC (*p* = 1.000). Finally, SRDC was chosen significantly more often than SPDC (*p* = 0.003). For the 7–8-year age group, SRSC was chosen significantly more often than SPDC (*p <* 0.001) and SRDC (*p* = 0.005), but did not differ significantly from TSPR (*p* = 0.326). TSPR was chosen significantly more than SPDC (*p* = 0.005); however, no difference was found between TSPR and SRDC (*p* = 1.000). Further, SPDC was chosen significantly less than any of the other three (all *p <* 0.001). For the 9–10-year age group, SRSC was chosen significantly more than SPDC (*p <* 0.001) and SRDC (*p <* 0.001); however, no difference was found between SRSC and TSPR (*p* = 1.000). TSPR was chosen significantly more than both SPDC (*p <* 0.001) and SRDC (*p =* 0.003). However, SRSC was chosen significantly less than SPDC (*p* = 0.002).

In conclusion, the results of Experiment 2 fully supported H2a–H2c. SRSC rates rose following the red highlighting (Δ 5–6 yrs = +4.4%; Δ 9–10 yrs = +10.4%), with the largest gains seen among the youngest group. The 7–8 and 9–10-year-olds no longer differed (*p* = 1.000). SRSC and TSPR were statistically equivalent (*p* = 1.000), whereas SPDC remained the least chosen.

### 4.5 Discussion

In the weak cue condition, the red semantic radical provided additional support for younger children. Among 9–10-year-olds, the higher rate of SRSC selections compared to TSPR suggests that children at this age have developed a basic understanding that semantic radicals encode category information. The 7–8-year age group appears to represent a turning point: at this stage, SRSC and TSPR were chosen at similar rates, but after this point children increasingly relied on semantic radicals for categorization. Compared with Experiment 1, Experiment 2 facilitated classification by emphasizing visual cues. In this experiment, selection rates were highest for SRSC and TSPR, conditions in which characters shared either a semantic or phonetic radical. This pattern suggests that visual similarity remains a key basis for category judgments in younger children. With age and schooling, however, children begin to grasp that semantic radicals convey categorical meaning, and visual cues help them refine their understanding of morphological pathways in Chinese characters. Under the weak cue condition, both the 7–8- and 9–10-year groups categorized more often according to semantic rather than phonetic radicals, whereas the 5–6-year group still showed a preference for phonetic radicals. Because the phonetic radical is typically the largest component of a character, it may constrain younger children’s reasoning. Around ages 6–7, as children transition from concrete to preoperational thinking, the weak cue condition can enhance their use of semantic radicals but cannot fully overcome these developmental limits.

## 5 Experiment 3: how the semantic radical facilitates the development of children’s inductive reasoning ability under the strong cue condition

### 5.1 Participants

Participants were recruited from private kindergartens and elementary schools in Kunming, Yunnan Province. As in the previous experiments, 45 participants from each age group were randomly selected, for a total of 135 participants. To match with Experiment 1, the final valid sample included 107 participants, including 34 children aged 5–6 years (mean age: 67.7 months, range: 62–72 months), 33 children aged 7–8 years (mean age: 92.2 months, range: 85–95 months), and 40 children aged 9–10 years (mean age: 116.2 months, range: 110–119 months). The study was approved by the appropriate ethics committee [blinded for review].

### 5.2 Materials

The visual materials used in Experiment 3 were identical to those in Experiment 2; however, the instructions given in Experiment 3 differed.

### 5.3 Procedures

The instructions were the same as in Experiment 1; however, when the researcher read an alternative word and asked the children to choose, the children were explicitly told that the red semantic radical could indicate the same category. Each child completed 40 trials.

### 5.4 Results

**FIGURE 6:**
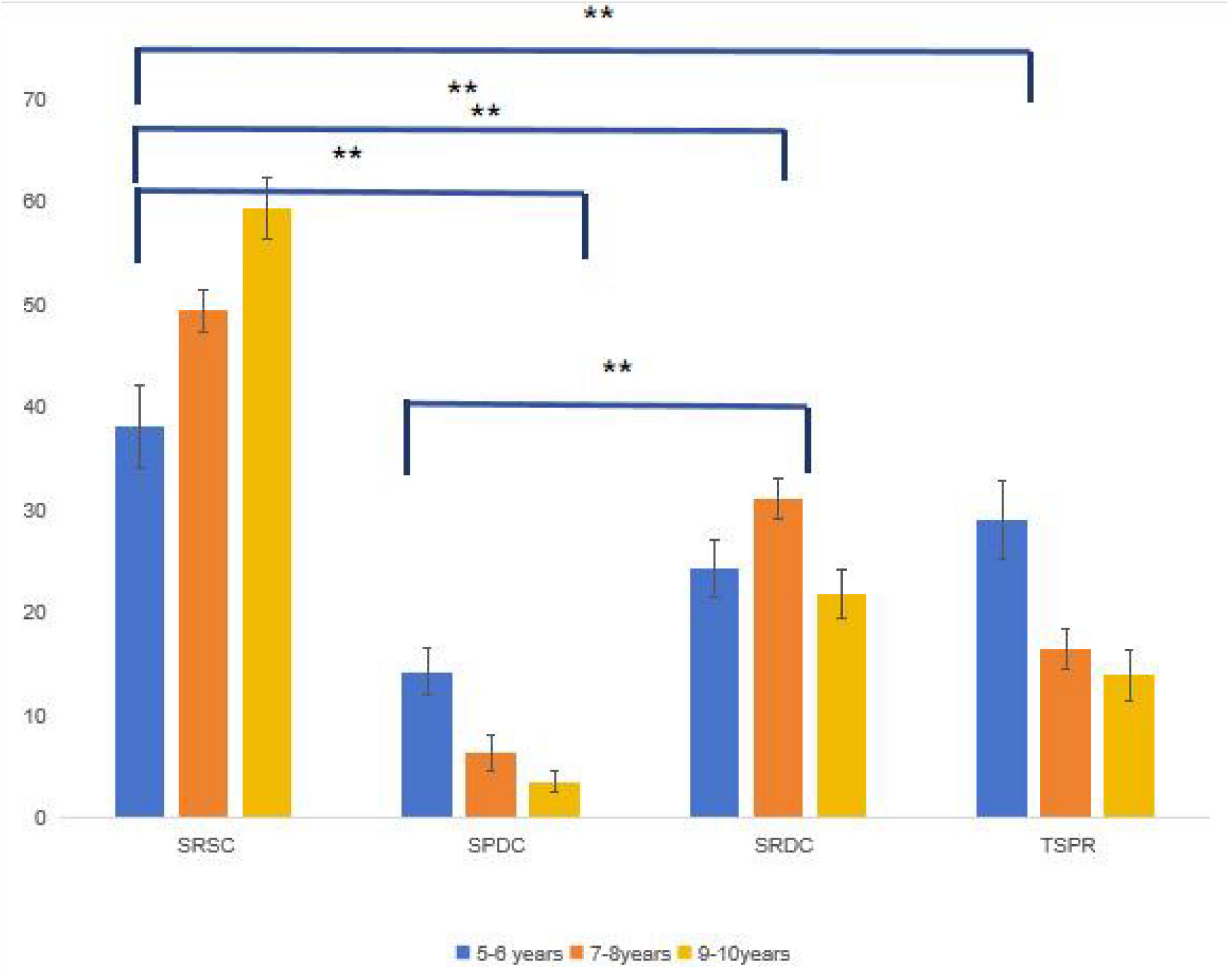
Selection rate in the strong cue condition *for *p <* 0.05, **for *p <* 0.01.

The results of Experiment 3 are shown in Table 4 and Figure 6.

**TABLE 4:** Results of the strong cue condition [M(SE)].

Cue condition	5–6 years	7–8 years	9–10 years
SRSC	38.164(3.995)	49.350(2.156)	59.274(2.964)
SPDC	14.284(2.218)	6.309(1.734)	3.548(1.084)
SRDC	24.287(2.796)	31.118(2.016)	21.827(2.371)
TSPR	28.994(3.833)	16.462(1.887)	13.939(2.435)

SRSC, same radical and meaning; SPDC, similar pronunciation but no category relationship with the target; SRDC, same radical but different category; TSPR, the same phonetic radical.

The relationship main effect was significant [*F*(3, 312) = 101.975, *p <* 0.001, η*_*p*_*^2^ = 0.495], and the *post-hoc* test (Bonferroni correction) showed that SRSC was chosen significantly more than any of the other three (all *p <* 0.001). SRDC was chosen the second most often out of the others, and it was chosen significantly more often than SPDC (*p <* 0.001). However, the difference between SRDC and TSPR was not significant (*p* = 0.056). SPDC was chosen the least often out of all the others (all *p <* 0.001). The age main effect was significant [*F*(3, 104) = 5.367, *p* = 0.006, η_*p*_^2^ = 0.094], and the *post-hoc* test (Bonferroni correction) showed a significant difference between the 5–6 and 9–10-year (*p* = 0.005) groups, but no significant difference between the 5–6 and 7–8-year (*p* = 0.866) groups. Additionally, there was no difference between the 7- and 8-year and 9–10-year (*p* = 0.866) groups.

The interaction of the relationship with age was significant [*F*(3, 312) = 8.888, *p <* 0.001, *η_*p*_*^2^ = 0.146]. Moreover, a simple effects analysis (for the effect of age under the different relationships) revealed that the main effect of age was significant for all four groups [SRSC: *F*(2, 104) = 11.665, *p <* 0.001; SPDC: *F*(2, 104) = 10.933, *p <* 0.001; SRDC: *F*(2, 104) = 3.884, *p* = 0.024; and TSPR: *F*(2, 104) = 8.121, *p* = 0.001]. In the SRSC condition, the 9–10-year group showed significantly higher selection ratios than the 5–6-year (*p* < 0.01) and 7–8-year (*p* = 0.049) groups; no significant difference was found between the 5–6- and 7–8-year groups (*p* = 0.079). The SPDC relationship showed that the selection rate exhibited a decreasing trend with age, with children aged 9—-10 years selecting this item at a significantly lower rate than children aged 5–6 years (*p* = 0.005). However, these findings were not significantly different from those for the comparison between the 7–8-year age group and the 5–6-year age group (*p* = 0.738)—children aged 7–8 years selected this item at a significantly lower rate than children aged 5—-6 years (*p* = 0.005). In the SRDC condition, the 7–8-year group showed the highest selection rate, which was significantly higher than that of the 9–10-year group (*p* = 0.023). However, the 7–8-year group did not differ significantly from the 5–6-year group (*p* = 0.169), and no significant difference was found between the 5–6- and 9–10-year groups (*p* = 1.000). There was a decreasing trend in the selection of TSPR with increasing age, with children aged 5—-6 years selecting this item at a significantly higher rate than children aged 7–8 years (*p* = 0.009) and 9—-10 years (*p* = 0.001). However, there was a non-significant difference between children aged 7–8 years and 9–10 years (*p* = 1.000).

A simple effects analysis (examining relationship type within each age group) showed significant main effects in all groups [5–6 years: *F*(3, 99) = 6.976, *p* = 0.001; 7–8 years: *F*(3, 96) = 71.593, *p* = 0.001; 9–10 years: *F*(3, 117) = 83.913, *p* = 0.001]. For the 5–6-year group, SRSC was chosen significantly more often than SPDC (*p* < 0.001), but not more often than SRDC (*p* = 0.136), and did not differ from TSPR (*p* = 1.000). TSPR was chosen significantly more often than SPDC (*p* = 0.029), but it did not differ from SRDC (*p* = 1.000). SRDC was chosen significantly more often than SPDC (*p* = 0.017). For the 7–8-year group, SRSC was chosen significantly more often than all three alternatives (all *p* < 0.001). SRDC was chosen significantly more often than SPDC (*p* < 0.001) and TSPR (*p* < 0.001). TSPR was chosen significantly more often than SPDC (*p* = 0.001). For the 9–10-year group, SRSC was chosen significantly more often than all three alternatives (all *p* < 0.001). SRDC was chosen significantly more often than SPDC (*p* < 0.001), but not more often than TSPR (*p* = 0.099). TSPR was chosen significantly more often than SPDC (*p* = 0.001).

In conclusion, in Experiment 3, all predictions were upheld. SRSC was significantly higher than in the two weaker cue conditions (*p* < 0.01). Further, 5–6-year-olds chose SRSC (38.2%) significantly more often than TSPR (29.0%), *p* < 0.001. The age effect was significant only between 9- and 10- and 5–6-year-olds (*p* = 0.005). In contrast, the choices of 5–6- and 7–8-year-olds did not differ significantly (*p* = 0.866). The relation-type order was completely reversed: SRSC dominated, and SPDC was the least selected (*p* < 0.001).

### 5.5 Discussion

The overall trend was a gradual increase in the rate of induction according to the same-semantic radical with age. This suggests that the role of linguistic labels can be modified through education. Educators may be able to enhance children’s categorization skills through conscious education. Even the youngest group, the 5–6-year-olds, showed a bias toward reasoning according to meaningful symbols when the experiment directly suggested a category relationship. There was still no significant difference between SRSC and TSPR among the 5–6-year age group, which may indicate that the induction in this age group was largely based on sensory similarity. This abrupt gain is consistent with Sloutsky and Napolitano’s (2003) finding that young children assign greater weight to auditory labels than to visual pictures; once the semantic radical was explicitly named as a category marker, the orally delivered label could override the otherwise dominant phonetic-visual pull and shift choices toward the SRSC option. Although a category relationship was provided, it was largely affected by the visual and phonetic similarities of the TSPR relationship.

A significant proportion of the subjects in Experiment 2 chose according to the same phonetic radicals, likely because these radicals typically occupy the largest portion of a character and often correspond to similar pronunciations. This suggests that children relied on sound–symbol agreement, integrating both visual and phonological similarity cues.

## 6 Analysis of the SRSC condition across age groups and different cue conditions

### 6.1 Results

The results are presented in Table 5.

**TABLE 5:** Results of the SRSC condition across age groups and different cue conditions [M(SE)].

Cue condition	5–6 years	7–8 years	9–10 years
No cue	20.319(2.63)	34.366(2.015)	32.352(3.073)
Weak cue	24.726(2.73)	38.887(1.814)	42.736(3.805)
Strong cue	38.164(3.995)	49.350(2.156)	59.274(2.964)

To analyze the developmental trend of the SRSC selection rate, we analyzed it across different age groups (see Table 4). Significant cue [*F*(2, 208) = 48.113, *p <* 0.01, *η_*p*_*^2^ = 0.316] and age [*F*(1, 104) = 17.645, *p <* 0.01, *η_*p*_*^2^ = 0.253] effects were observed. The interaction of the cue and age effects was not significant [*F*(4, 208) = 1.622, *p* = .170, *η_*p*_^2^* = 0.03]. The Bonferroni-corrected *post-hoc* test revealed that the choice of SRSC was significantly improved by cue strength. Strong cues scored significantly higher than either weak (*p <* 0.01) or no (*p <* 0.01) cues. Further, weak cues scored significantly higher than no cues (*p* = 0.05). The Bonferroni-corrected *post-hoc* test for age showed that the 9–10 age group scored significantly higher than the 5–6 group (*p <* 0.01), but not the 7–8 group (*p* = 0.577). Further, the 7–8 age group scored significantly higher than the –6 group.

### 6.2 Discussion

The results of this study provide clear evidence for the impact of cue strength on selection. The strong cue condition yielded significantly higher scores than both the weak and no cue conditions, suggesting that the presence and strength of cues significantly influenced the accuracy of SRSC selection. This finding is consistent with the principle that semantic radicals may provide an additional “morphosemantic” pathway from the radical form to the category to which they belong.

The age effect was also significant, with the 9–10 age group outperforming younger age groups. This suggests that, as children age, their orthographic awareness improves, which may be attributed to the maturation of cognitive abilities and increased metalinguistic awareness. However, the non-significant interaction between cue effect and age indicated that the influence of cue strength did not significantly differ across age groups. This could imply that age, as a factor of maturation, and cue strength, as a factor of acquired experience, independently contribute to the categorization process in children.

## 7 General discussion

The present study addressed the three theoretical questions outlined in the introduction:

(a)    Is the label effect driven by conceptual or similarity-based information?
(b)    What is the relative weight given to visual versus auditory inputs during children’s induction?
(c)    What is the exact age at which the developmental shift from similarity- to category-based reasoning occurs?

By manipulating the semantic and phonetic radicals of picto-phonetic characters, three experiments systematically examined how 5–10-year-old children perform inductive inference in a Chinese character context. The findings provided the following answers.

The label effect is not uniform; rather, it is dynamically shaped by cue strength and age. In the no-cue condition, children predominantly relied on the phonetic radical (TSPR), suggesting that perceptual similarity governs early induction. By contrast, when the semantic radical was highlighted or explicitly explained, selections of the same-category radical (SRSC) increased sharply, indicating that external semantic cues can effectively activate a conceptual route. These findings support the dual-path view of linguistic labels (Gelman and Davidson, 2013; Wang et al., 2018) and further demonstrate that within pictophonetic characters, the semantic radical serves as an embedded label guiding categorical inference even without whole-character recognition (Zhang et al., 2014). In addition echoed by Mari et al. (2023), who showed that both linguistic labels and statistical evidence jointly guide 6–10-year-olds’ inferences about novel social categories, with older children weighting category labels more heavily than younger children. This cross-domain convergence underscores that the developmental shift from perceptual- to label-based induction is not restricted to orthographic materials but generalizes to social-categorical learning as well.”

Second, the visual channel has a natural advantage in Chinese character processing. Although some studies have reported auditory dominance in young children (Napolitano and Sloutsky, 2010), we found that even 5–6-year-olds preferred the phonetic radical (TSPR), which offers both visual and phonological overlap, over the SPDC condition, which has only phonological similarity. More importantly, the semantic radical, a visual component, successfully directed categorical responses once cued, indicating that visual information can be educationally channeled into conceptual processing. This “visually augmented induction” highlights the unique morphosemantic pathway of Chinese reading (Pae, 2020; Zhang et al., 2023).

Ages 7–8 represent a critical turning point, this conceptual shift is consistent with findings that older children and adults place greater weight on the causal–mechanistic links among features than on sheer perceptual overlap when making category-based inferences (Rehder and Burnett, 2005). Viewed in this light, the semantic radical can be construed as a miniature “causal cue”: its presence signals that the character is generated by the same underlying category mechanism, thereby licensing the generalization of hidden properties—a strategy that becomes increasingly dominant after 7–8 years of age.” In the no-cue condition, their response patterns fell between those of 5–6- and 9–10-year-olds, but in the weak- and strong-cue conditions, their performance quickly converged with that of the older group. This suggests that 7–8-year-olds possess emerging radical awareness but still require external support to override perceptually driven choices. Such a shift aligns with the documented maturation of inhibitory control and cognitive flexibility during this period (Davidson et al., 2006; Zelazo et al., 2003). Rather than an abrupt replacement of perceptual by conceptual strategies, the present data fit an overlapping-waves account (Siegler, 2016): Phonetic, visual-only, and semantic-radical strategies co-exist across ages, but their relative frequencies shift as orthographic experience and metalinguistic control accumulate. Continuous instructional emphasis on semantic radicals can therefore further tip the distribution toward category-based induction even beyond the age ranges examined here.

Taken together, our results provide the first experimental evidence that semantic radicals function as embedded category labels, with inductive utility that varies by cue strength and age. We propose that the semantic radical is not merely structural but also a cognitive tool through which children construct early semantic systems. Systematic instruction that highlights radicals may therefore foster categorical and scientific thinking in the lower primary grades.

This study has several limitations. First, character frequency, stroke number, and other orthographic variables were not rigorously controlled; future work should incorporate these as covariates in mixed-effects models. Second, the task relied solely on auditory presentation, and visual or picture–word formats could further test modality preferences. Third, individual differences in executive function and language ability were not assessed; standardized measures will be needed to clarify how these factors moderate radical use.

Before closing, it is worth recalling that different languages share common origins: both Chinese characters and the English alphabet began as object-based depictions but evolved along divergent paths. Archeological evidence shows that the earliest direct ancestors of the modern English alphabet—the Proto-Sinaitic inscriptions from the Sinai Peninsula, dating to the nineteenth century BCE—were pictographic-phonetic symbols devised by Canaanite miners, adapted from Egyptian hieroglyphs through the acrophonic principle, and marking the transition from pictographic to alphabetic writing (Powell, 1991). In contrast, Chinese has retained more graphic information, making its morphology-to-meaning processing pathway more prominent. Neuroimaging studies using near-infrared spectroscopy further demonstrate that Chinese reading relies more heavily on morpho-semantic processing, whereas English reading emphasizes phonological processing; consequently, the neural activation patterns of the two languages are complementary yet asymmetrical (Zhang et al., 2023).

However, this hypothesis must be tested in future studies. All characters in this study were left–right structured pictophonetic characters. Although these are the most common type, top–bottom structures also exist and should be included in future materials to clarify the role of semantic radicals in inductive categorization. Future work could also employ linear mixed-effects models with random intercepts for participants and items, while incorporating covariates such as character-reading vocabulary or receptive vocabulary scores. This approach would help reduce literacy-related heterogeneity and quantify how individual differences modulate the Radical Cue × Age interaction, yielding a more precise estimate of the cross-sectional developmental pattern.

It should be noted that the present study did not directly measure or statistically control for individual differences in verbal ability or domain-general inductive reasoning (e.g., non-verbal analogical reasoning). Nevertheless, all children aged 7–10 years were recruited from the same primary school, and all 5–6-year-olds were recruited from the same kindergarten. As such, all participants followed a similar standard curriculum. Consequently, extreme disparities in language or reasoning proficiency are unlikely. Nevertheless, future research should include standardized vocabulary or general reasoning scores to reduce residual heterogeneity and to clarify the specific contribution of orthographic awareness to inductive reasoning in Chinese-learning children.

In summary, in the present study, participants may have shifted from reasoning based on perceptual similarities to reasoning based on concepts, which may have been due to their educational experience. Ultimately, as children develop orthographic awareness and literacy, they can better understand the link between semantic radicals and the categories of pictophonetic Chinese characters, enabling them to reason according to these categorical relationships.

## Data Availability

The raw data supporting the conclusions of this article will be made available by the authors, without undue reservation.
